# Peristaltic Phenomenon in an Asymmetric Channel Subject to Inclined Magnetic Force and Porous Space

**DOI:** 10.3390/bioengineering9100588

**Published:** 2022-10-20

**Authors:** Muhammad Ijaz Khan, Maha M. A. Lashin, Nidhal Ben Khedher, Bilal Ahmed, Sami Ullah Khan, Mowffaq Oreijah, Kamel Guedri, El Sayed Mohamed Tag-ElDin, Ahmed M. Galal

**Affiliations:** 1 Department of Mathematics and Statistics, Riphah International University, Islamabad 44000, Pakistan; 2 College of Engineering, Princess Nourah Bint Abdulrahman University, Riyadh 11564, Saudi Arabia; 3 Department of Mechanical Engineering, College of Engineering, University of Ha’il, Ha’il 81451, Saudi Arabia; 4 Laboratory of Thermal and Energy Systems Studies, National School of Engineering of Monastir, University of Monastir, Monastir 5000, Tunisia; 5 Department of Mathematics and Statistics, The University of Lahore, Sargodha Campusi, Punjab 40100, Pakistan; 6 Department of Mathematics, COMSATS University Islamabad, Sahiwal 57000, Pakistan; 7 Mechanical Engineering Department, College of Engineering and Islamic Architecture, Umm Al-Qura University, Makkah 21955, Saudi Arabia; 8 Research Unity: Materials, Energy and Renewable Energies, Faculty of Science of Gafsa, University of Gafsa, Gafsa 2100, Tunisia; 9 Faculty of Engineering and Technology, Future University in Egypt, New Cairo 11835, Egypt; 10 Department of Mechanical Engineering, College of Engineering in Wadi Alddawasir, Prince Sattam Bin Abdulaziz University, Al-Kharj 16278, Saudi Arabia; 11 Production Engineering and Mechanical Design Department, Faculty of Engineering, Mansoura University, Mansoura 35516, Egypt

**Keywords:** peristaltic flow, non-zero Reynolds number, asymmetric channel, finite element method, porous media

## Abstract

This research is engaged to explore biological peristaltic transport under the action of an externally applied magnetic field passing through an asymmetric channel which is saturated with porous media. The set of governing partial differential equations for the present peristaltic flow are solved in the absence of a low Reynolds number and long wavelength assumptions. The governing equations are to be solved completely, so that inertial effects can be studied. The numerical simulations and results are obtained by the help of a finite element method based on quadratic six-noded triangular elements equipped with a Galerkin residual procedure. The inertial effects and effects of other pertinent parameters are discussed by plotting graphs based on a finite element (FEM) solution. Trapped bolus is discussed using the graphs of streamlines. The obtained results are also compared with the results given in the literature which are highly convergent. It is concluded that velocity and the number of boluses is enhanced by an increase in Hartmann number and porosity parameter K Increasing inertial forces increase the velocity of flow but increasing values of the porosity parameter lead to a decrease in the pressure gradient. The study elaborates that magnetic field and porosity are useful tools to control the velocity, pressure, and boluses in the peristaltic flow pattern.

## 1. Introduction

The phenomenon in which fluid moves along the axis of a channel due to the contraction and relaxation of the walls is called peristalsis. Many scientists and engineers have been working on peristaltic flow due its interesting applications. Which involves discussion of Newtonian and non-Newtonian flows. The peristaltic flow of non-Newtonian fluids is currently an emerging field. Many applications of these phenomena have been seen in the engineering and bio-medical fields. Peristaltic flow also involved in human and animal organs, such as the flow of urine from the kidneys, blood flow to and from the heart, and the movement of chyme (partially digested food). Devices used in the printing industry, peristaltic pumps used in the chemical industry for manufacturing strong acids, and bio-medical devices work under the principle of peristalsis. Peristalsis is an emerging field which has attracted researchers to work on it. The first research took place in 1964, when Latham [1] worked on a peristaltic pump and demonstrated the peristaltic flow in the channel. He investigated peristalsis in a fixed frame of reference. After that, Shapiro et al. [2] studied the peristalsis phenomenon in a channel which was two-dimensional by geometry, but they used the assumptions of low lubrication theory which hides many inertial effects which should be discussed. They also presented theoretical results for both planes. Fung and Yih [3] investigated peristalsis in an infinite channel. In their investigation it was seen that peristaltic pumping produces reverse flow when its value is increased to a critical value, and they concluded that velocity profile is dependent on pressure gradient. They also observed that convergence of results decreases when the value of Reynolds numbers is too large. In an investigation by Hanin [4], large Reynolds numbers and a small wavelength were used. Zein and Ostrach [5] analyzed peristaltic phenomena for two-dimensional geometry with the limitations of long wavelength and low frequency. They obtained the solution in closed form and compared it with the result of Fung and Yih [3]. Peristaltic flow in tubes was also discussed by Barton and Raynor [6], but long-wave and short-wave disturbances were only discussed for Stokes flow. Chow [7] investigated peristaltic flow by employing long wavelength approximations. Peristaltic flow was also discussed in detail by Brown and Hung [8], who solved the governing equation with the help of a finite difference technique. Jaffrin [9] studied peristalsis in a two-dimensional tube by using a perturbation technique with the employment of non-linear effects. The detailed work on peristaltic phenomena in the channel by using the finite difference method was elaborated by Takabatake and Ayukawa [10]. They considered the complete set of equations that governs the problem. They examined the model with moderate values of Reynolds numbers and made a comparison with the results of perturbation analysis. An investigation on the full form of partial differential equations for peristaltic transport in the tube was also carried out by Takabatake et al. [11]. The trapping of bolus was also taken into account in their approach for studying peristaltic maxing. It is observed that pumping efficiency is greater in tubes in comparison with flat two-dimensional channels. Takabatake et al. [12] analyzed and studied the work which was undertaken earlier by Takabatake et al. [11] by using the finite element method (FEM), and compared the results with those obtained in the work of Dennis and Chang [13]. Takabatake [14] investigated pressure rise per wavelength by engaging the finite element method (FEM), and a comparison of the results with results built on perturbation analysis was also given in their study. Bhatti et al. [15] elaborated the peristaltic activity for Prandtl nanofluid transport with endoscopic annular geometry. Zeeshan et al. [16] focused on aspects of Jeffrey fluid flow within peristalsis. The determination of Prandtl nanoparticles following the peristaltic pattern was noticed in the framework of Abbasi et al. [17]. Riaz et al. [18] identified the thermal role of nano-sized particles with the slip flow problem due to peristaltic motion. Le et al. [19] pointed out the inertial consequences for the inclined channel flow due to peristaltic transport.

During the last few decades researchers and scientists have worked on peristaltic flow through porous media due to its wide range of application in various fields. Movement of fluid in plants and in the human body are examples of flow through porous media. Mining of rudimentary oil from the earth also involves a porous medium. What we mean by porous medium is something that includes minor holes over its whole surface which allow fluid to flow through it. Examples of porous media are sand, limestone, or a loaf of bakery bread. In living things such as humans, the gallbladder, kidney, and lungs are porous media. Hayat et al. [19] worked on peristaltic transport of Maxwell fluid which conducts electrically through a channel soaked with porous media. They found the solutions analytically and discussed the situations graphically. It was observed that mean velocity decreases with the increment of the Hall parameter. Srinivas and Kothandapani [20] found the analytical solution for peristaltic transport through a porous medium with the application of lubrication theory, which makes the governing equations very simple to discuss. It is seen that with an increase in the Schmidt number, the concentration decreases. Tripathi and Bég [21] investigated oscillating peristaltic flow of generalized Maxwell fluid through a porous medium by employing lubrication theory. They used the homotopy perturbation method to obtain the approximate analytical and numerical solutions. It was detected that the volumetric flow rate decreased when the relaxation time increased. Ellahi et al. [22] worked on the theoretical study of MHD effects on peristaltic flow in a rectangular duct through a porous medium employing long wavelength and low Reynolds number assumptions, but the solutions were obtained analytically. Trapping of bolus and the effects of MHD were also discussed. Sankad and Nagathan [23] studied MHD effects on peristaltic flow through porous media; the slip condition’s effects were also taken into account and solutions were obtained analytically. The proposed results were discussed with the help of graphs. They modeled the governing equation by using low Reynolds number and long wavelength assumptions. It was observed that mean velocity rises with the magnetic field parameter in the presence of viscous damping force, but opposite behavior was seen when this force was removed.

In the present era, scientists are taking much interest in the discussion of fluid flowing through a channel or wall under the presence of an external magnetic force due to the number of applications in industry and astrophysics, etc., and its thought-provoking features. Magnetohydrodynamics (MHD) is very powerful tool in the medical field which is used in the diagnosis of hypothermia, tumors and MRI. The investigation of MHD peristaltic phenomena of a Newtonian fluid in a channel was presented by Yildirim and Sezer [24]. They obtained the solutions analytically and numerically by applying the homotopy perturbation method with low Reynolds number and long wavelength assumptions and made a comparison with the results of Nadeem and Akram [25]. It was seen that with an increase in L and M the temperature decreases. Nadeem and Akbar [26] studied MHD peristaltic flow of Newtonian fluid and discussed the effect of radially changing MHD. In the analysis, assumptions of lubrication theory were applied to obtained simplified governing equations, to facilitate ease of discussion. Manzoor el al. [27] discussed low-pressure plasma on degradation of multidrug resistant V. Cholera with antibacterial utilizations. Chu et al. [28] examined four different types of nanomaterials with thermal radiation and non-linear heat source and sink. Ramesh and Devaker [29] studied the peristaltic flow in porous asymmetric channel with the effects of external magnetic field and heat transfer. It is seen in the analysis that the pumping is increased at higher Hartmann numbers.

By the use of assumptions of low Reynolds numbers and long wavelength, the partial differential equations become very simple to discuss and hide many inertial effects which must be the part of discussion. To overcome this problem, Javed et al. [30] presented work on peristaltic flow in a two-dimensional inclined channel, employing an external magnetic field and without the use of lubrication theory. Many important inertial effects are discussed in their analysis; for example, they concluded that bolus size increased with increasing Reynolds numbers. The effects of Reynolds numbers on vorticity were also presented in their analysis. Ahmed at el. [31] also investigated peristaltic phenomena without employing low Reynolds number and long wavelength assumptions in a vertical channel. During analysis they concluded that heat generation controls the velocity, i.e., a rise in heat generation diminishes the velocity near the central region of the channel, and opposite behavior was observed near the walls. The peristaltic phenomena without the use of lubrication theory were also presented by Javed et al. [32]. They used the finite element method to obtain the numerical results. It was seen that the presented effects of Reynolds number are true for arbitrary values. Recently, some dynamic researchers investigated peristaltic flow [30,31,32,33] without engaging lubrication theory’s assumptions about the channel, which is symmetric around its axis. In the literature, it is seen that many researchers have used lubrication theory, which leads the sets of partial differential equations that govern the peristaltic flow to be very simple to solve; however, those results leads to the exclusion of inertial terms in the discussion. As a result of this, the inertial effects and other significant aspects have not been studied. Some studies [34,35,36,37,38] interpret some basic concepts on fluid flow and nanomaterials.

The aim of the current investigation is to observe the peristaltic phenomenon in an asymmetric channel saturated with porous media, along with the applications of external orthogonal magnetic field effects without engaging the norms of low Reynolds numbers and long wavelengths, which causes the system to be non-linear. The governing equations will be solved in full form using the finite element method equipped with Galerkin’s residual approach by using triangular elements in meshing with six nodes for numerical solution, which is highly convergent in comparison with linear triangular elements. Moreover, solving the system of equations in full form enables us to study the inertial effects and many other parameters on peristaltic flow. Besides this velocity distribution, pressure evaluation and trapping activity will be discussed with the help of graphs plotted based on numerical results.

## 2. Problem Formulation

**Figure 1:**
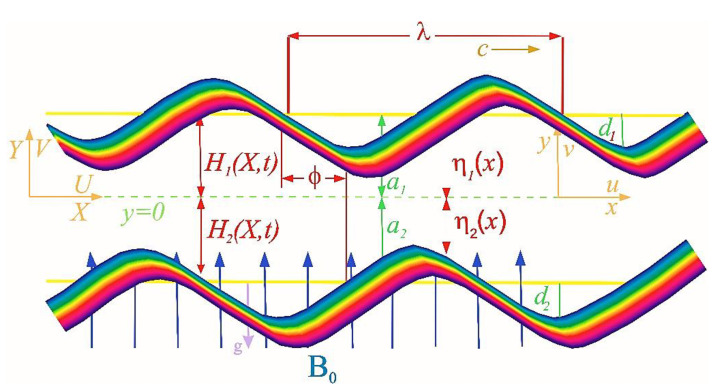
Geometry of the problem.

In our present work we are considering an unsteady incompressible flow through a channel soaked with porous media. The considered flow is moving through a two-dimensional asymmetric channel of width 2a lying horizontally. Further, we have applied an external magnetic field of strength $B_{0}$ orthogonal to the direction of the flow. The fluid is a conductor electrically; due to this, an induced magnetic field arises, but it is insignificant in comparison with our applied $B_{0}$. Thus, we consider only the effect of the external magnetic field. The fluid flows due to the wave generated on the walls of the channel. The geometry of our considered problem is given in Figure 1. The upper moving wall of the infinite channel follows the relation given by [9,10]:

(1) $$H_{1}\left(X,t\right)=a_{1}+b_{1}\cos\left(\frac{2\pi \left(X-ct\right)}{\lambda }\right),$$

The lower walls obey the relation given by

(2) $$H_{2}\left(X,t\right)=-a_{2}-b_{2}\cos\left(\frac{2\pi \left(X-ct\right)}{\lambda }+\phi \right)$$

In the above relations, $b_{1}$ and $b_{2}$ represents the amplitudes, λ represents the wavelength of the wave, c is the speed of the wave, 2a is the width of the channel ($a_{1}=a_{2}=a$)*,* and ϕ is the phase difference with (0<ϕ≤π). Note that when ϕ=0 the corresponding channel is symmetric with the waves out of phase and when ϕ=π the corresponding channel is symmetric and the waves are in phase. Furthermore, $a_{1},a_{2},\;b_{1},b_{2}\;\mathrm{and}\;\phi$ fulfil the requirements of the inlet of the divergent channel, i.e.,

(3) $$a_{1}^{2}+a_{2}^{2}+2b_{1}b_{2}\cos\phi \leq {\left(a_{1}+a_{2}\right)}^{2}$$

The governing equations for our present problem, i.e., the principles of conservation of momentum and conservation of mass and the law of for conducting fluid through a channel which is saturated with porous media under the magnetic field in fixed frame are expressed as [9,10]:

(4) $$\nabla \times V^{*}=0,$$

(5) $$\rho \left(V^{*}\times \nabla V^{*}\right)=-\nabla P^{*}+\mu \left(\nabla^{2}V^{*}\right)+\left(J\times B\right)-\frac{\mu }{k}V^{*}.$$

In the above equations, $V^{*}=\left(U,V,0\right)$ is the velocity of fluid in two dimensions, and J and B are the current density and magnetic field, respectively. As the magnetic field is applied orthogonally, $B=\left(0,\;B_{0},\;0\right)$, ρ is the density, μ is the coefficient of dynamic viscosity, and k is the permeability of the porous media. The Lorentz force after neglecting the induced magnetic field (very small as compared with the external magnetic field) is given by

(6) $$J\times B=\left[-\sigma B_{0}^{2}U,\;0,\;0\right]$$

The Equations (4) and (5) in a fixed frame take the form [9,10]:

(7) $$\frac{\partial U}{\partial X}+\frac{\partial V}{\partial Y}=0,\;$$

(8) $$\rho \left\{\frac{\partial U}{\partial t}+U\frac{\partial U}{\partial X}+V\frac{\partial U}{\partial Y}\right\}=-\frac{\partial P}{\partial X}+\mu \left(\frac{\partial^{2}U}{\partial X^{2}}+\frac{\partial^{2}U}{\partial Y^{2}}\right)-\sigma B_{0}^{2}U-\frac{\mu }{k}U,$$

(9) $$\rho \left\{\frac{\partial V}{\partial t}+U\frac{\partial V}{\partial X}+V\frac{\partial V}{\partial Y}\right\}=-\frac{\partial P}{\partial Y}+\mu \left(\frac{\partial^{2}V}{\partial X^{2}}+\frac{\partial^{2}V}{\partial Y^{2}}\right)-\frac{\mu }{k}V,$$

with suitable boundary conditions given as

(10) $$\;U=0,\;\;\;\;\;\;\;\;\;\;\;\;\;\;\;\;\;\;\;\;\;\;\;\;\;\;\;\;V=\frac{\partial H_{1}}{\partial t},\;\;\;\;\;\;\;\;\;\;\;\;\;\;\;\;\;\;\;\;\;\;at\;\;\;\;\;\;\;\;\;\;Y=H_{1}\left(X,t\right),$$

(11) $$U=0,\;\;\;\;\;\;\;\;\;\;\;\;\;\;\;\;\;\;\;\;\;\;\;\;\;\;\;\;V=\frac{\partial H_{2}}{\partial t},\;\;\;\;\;\;\;\;\;\;\;\;\;\;\;\;\;\;\;\;\;\;at\;\;\;\;\;\;\;\;\;\;Y=H_{2}\left(X,t\right).$$

The conditions on U are due to no-slip conditions on the walls and the boundary conditions on V mean the wall velocity is same as the normal velocity of the walls. Now we transform our governing equations in a moving frame of reference $\left(x^{*},\;y^{*}\right);$ for this, both frames of references are related, as

(12) $$x^{*}=X-ct,\;\;\;\;\;\;\;y^{*}=Y,\;\;\;\;\;\;\;\;\;u^{*}=U-c,\;\;\;\;\;\;v^{*}=V,\;\;\;\;\;\;\;\;p^{*}=P,$$

where (U, V) and $\left(u^{*},v^{*}\right)$ are velocity components in the laboratory frame and wave frame, respectively. $p^{*}$ and P are pressures in the laboratory frame and wave frame. The governing equations after transformation in the moving frame using Equation (12) take the form:

(13) $$\frac{\partial v^{*}}{\partial y^{*}}+\frac{\partial u^{*}}{\partial x^{*}}=0,\;$$

(14) $$\rho \left\{v^{*}\frac{\partial u^{*}}{\partial y^{*}}+u^{*}\frac{\partial u^{*}}{\partial x^{*}}\right\}=-\frac{\partial p^{*}}{\partial x^{*}}+\mu \left(\frac{\partial^{2}u^{*}}{\partial x^{*}{}^{2}}+\frac{\partial^{2}u^{*}}{\partial y^{*}{}^{2}}\right)-\left(\sigma B_{0}^{2}+\frac{\mu }{k}\right)\left(u^{*}+c\right),$$

(15) $$\rho \left\{\frac{\partial v^{*}}{\partial x^{*}}u^{*}+\frac{\partial v^{*}}{\partial y^{*}}v^{*}\right\}=-\frac{\partial p^{*}}{\partial y^{*}}+\mu \left(\frac{\partial^{2}v^{*}}{\partial x^{*}{}^{2}}+\frac{\partial^{2}v^{*}}{\partial y^{*}{}^{2}}\right)-\frac{\mu }{k}v^{*},$$

with appropriate boundary conditions,

(16) $$\;\;\;\;\;\;\;\;\;\;\;\;\;\;\;\;u^{*}=-c,v^{*}=\frac{2\pi cb_{1}}{\lambda }\sin\left(\frac{2\pi x^{*}}{\lambda }\right),\;\;\;\;\;\;\;\;\;\;\;\;\;\;\;\;\;\;\;\;\;\;at\;\;\;\;\;y^{*}=\eta_{1}\left(x^{*}\right)$$

(17) $$u^{*}=-c,v^{*}=-\frac{2\pi cb_{2}}{\lambda }\sin\left(\frac{2\pi x^{*}}{\lambda }+\phi \right),\;\;\;\;\;\;\;\;\;\;\;\;\;at\;\;\;\;\;y^{*}=\eta_{2}\left(x^{*}\right),$$

where $\eta_{1}\left(x^{*}\right)$ represents the constraint on the upper peristaltic wall and $\eta_{2}\left(x^{*}\right)$ on the lower walls in the moving frame. The planes $y^{*}=\eta_{1}\left(x^{*}\right)$ and $y^{*}=\eta_{2}\left(x^{*}\right)$ follow the streamlines’ pattern and the current flow rate $q^{*}$ is constant throughout the cross section of the peristaltic channel in the moving reference frame. The consistent boundary conditions relating stream function are expressed as

(18) $$\psi^{*}=q^{*},\;\;\;\;\;\;\;\;\;\;\;\;\;\;at\;\;\;\;\;y^{*}=\eta_{1}\left(x^{*}\right)\;\;\;\;\;and\;\;\;\;\;\;\;\;y^{*}=\eta_{2}(x^{*}).$$

$\psi^{*}$ is the illustration of stream function and $q^{*}=Q^{*}-ca$ narrates the flow rates in the fixed frame and in the moving frame of reference. Two proposed dimensionless variables are given, by which we can make our differential equations dimensionless,

(19) $$x=\frac{x^{*}}{\lambda },\;\;\;\;\;\;\;\;\;\;\;\;\;\;y=\frac{y^{*}}{a_{1}},\;\;\;\;\;\;\;\;\;\;\;\;u=\frac{u^{*}}{c},\;\;\;\;\;\;\;\;\;\;\;v=\frac{v^{*}}{c},\;\;\;\;\;\;\;\;\;a=\frac{a_{2}}{a_{1}},\;\;\;\;\;\;\;\;b=\frac{b_{1}}{a_{1}},\;\bar{\;b}=\frac{b_{2}}{a_{1}},\;\;\;\;\;\;\;\;\;\;\;\;\;\;\;\eta_{1}=\frac{\eta_{1}^{*}}{a_{1}},\;\;\;\;\;\;\;\;\;\eta_{2}=\frac{\eta_{2}^{*}}{a_{1}},\;\;\;\;\;\;\;\;\alpha =\frac{a_{1}}{\lambda },\;q=\frac{q^{*}}{ca_{1}},\;\;\;\;\;\;Re=\frac{ca_{1}}{\nu }\alpha ,\;\;\;\;\;\;\;M=\sqrt{\frac{\sigma }{\mu }}B_{0}a_{1},\;\;\;\;\;K=\frac{k}{a_{1}^{2}},\;\;\;\;\;\;\;\;\;\;p=\frac{a_{1}^{2}p^{*}\left(x^{*}\right)}{c\;\lambda \mu }.$$

After making the dimensionless form of equations that governs the peristaltic phenomena and corresponding boundary conditions, we have

(20) $$\alpha \frac{\partial u}{\partial x}+\frac{\partial v}{\partial y}=0,\;$$

(21) $$Re\left\{\alpha u\frac{\partial u}{\partial x}+v\frac{\partial u}{\partial y}\right\}=-\alpha \frac{\partial p}{\partial x}+\alpha \left(\alpha^{2}\frac{\partial^{2}u}{\partial x^{2}}+\frac{\partial^{2}u}{\partial y^{2}}\right)-\left(\alpha M^{2}+\frac{\alpha }{K}\right)\left(u+1\right)$$

(22) $$Re\left\{\alpha u\frac{\partial v}{\partial x}+v\frac{\partial v}{\partial y}\right\}=-\frac{\partial p}{\partial y}+\alpha \left(\alpha^{2}\frac{\partial^{2}v}{\partial x^{2}}+\frac{\partial^{2}v}{\partial y^{2}}\right)-\frac{\alpha }{K}v,$$

(23) $$u=-1,v=2\pi b\alpha \;\sin\left(2\pi x\right),\;\;\;\;\;\;\;\;\;\;\;\;\;\;\;\;\;at\;\;\;\;\;y=\eta_{1}\left(x\right)=1+b\cos\left(2\pi x\right)$$

(24) $$u=-1,v=-2\pi \bar{b}\;\alpha \;\sin\left(2\pi x+\phi \;\right),\;\;\;\;at\;\;\;\;\;y=\eta_{2}\left(x\right)=-a-\bar{b}\cos\left(2\pi x+\phi \right)$$

where Re is the ratio of inertial and viscous forces known as the Reynolds number, Fr represents the Forhheimer number ($Fr=\frac{c^{2}}{ga}$), and K is the permeability of the porous medium. After elimination of pressure terms by taking derivative Equation (21) with respect to y and Equation (22) with respect to x and simplifying by inserting the stream function and vorticity given by

(25) $$u=\frac{\partial \psi }{\partial y},\;\;\;\;\;\;\;\;\;\;\;\;\;\;\;\;\;\;\;v=-\alpha \frac{\partial \psi }{\partial x},\;\;\;\;\;\;\;\;\;\;\;\;\;\;\;\;\;\;\;\;\omega =\left(\alpha \frac{\partial v}{\partial x}-\frac{\partial u}{\partial y}\right),$$

the governing equations in ψ−ω formulation take the form

(26) $$\left(\alpha^{2}\frac{\partial^{2}\psi }{\partial x^{2}}+\frac{\partial^{2}\psi }{\partial y^{2}}\right)=\;-\omega ,$$

(27) $$Re\left\{\frac{\partial \psi }{\partial y}\frac{\partial \omega }{\partial x}-\frac{\partial \psi }{\partial x}\frac{\partial \omega }{\partial y}\right\}=\nabla^{2}\omega -\frac{\omega }{K}+M^{2}\frac{\partial^{2}\psi }{\partial y^{2}}.$$

The proposed boundary conditions in Equations (18), (23), and (24) become

(28) $$\;\psi =q,\;\;\;\;\;\;\;\;\;\;\;\;\;\frac{\partial \psi }{\partial y}=-1,\;\;\;\;\;\;\;\;\;\;\frac{\partial \psi }{\partial x}=-2\pi bsin\left(2\pi x\right),\;\;\;\;\;\;\;\;\;\;\;\;\;\;\;\;\;at\;\;\;y=\eta_{1}\left(x\right)$$

(29) $$\psi =q,\;\;\;\;\;\;\;\;\;\;\;\frac{\partial \psi }{\partial y}=-1,\;\;\;\;\;\;\;\;\;\;\frac{\partial \psi }{\partial x}=-2\pi \bar{b}sin\left(2\pi x+\phi \right),\;\;\;\;\;\;\;at\;\;\;y=\eta_{2}\left(x\right),$$

where $\nabla^{2}=\alpha^{2}\frac{\partial^{2}}{\partial x^{2}}+\frac{\partial^{2}}{\partial y^{2}}$. It is necessary to note that by employing assumptions of lubrication theory, Equation (26) vanishes and Equation (27) becomes

(30) $$\frac{\partial^{4}\psi }{\partial y^{4}}+M^{2}\frac{\partial^{4}\psi }{\partial y^{4}}=0.$$

## 3. Finite Element Analysis

In this section we are going to obtain the numerical solution to partial differential equations that govern the problem. For this purpose the partial differential Equations (26) and (27) along with the proposed boundary conditions given in Equations (28) and (29) are considered to be solved by applying finite element method to obtained numerical solution. Using FEM, we can easily handle the complex domains and they have rapid convergence. The discretization technique used in the finite element method makes it more effective to solve non-linear problems. In our present problem, our aim is to solve the given governing equations in a comprehensive form so that the effects of Reynolds numbers and wave numbers can be discussed. For this, we discretize the domain into a non-uniform mesh based on triangular elements (quadratic) founded on six nodes using pdetool in MATLAB. Firstly, we find the solution on every element of the domain, then assemble all elements into a large matrix known as the global matrix. The resultant equations are a non-linear system of algebraic equations. Then we will use the Newton Raphson method to obtain the solution to the equations. The finite element method will be implemented as follows:

The nodal variables are approximated as

(31) $$\psi =\sum_{k=1}^{n}N_{k}\psi_{k},\;\;\;\;\;\;\;\;\;\;\;\;\;\;\;\;\;\;\;\;\;\;\omega =\sum_{k=1}^{n}N_{k}\omega_{k}$$

where $\psi_{k}$ and $\omega_{k}$ denote the nodal approximation of ψ and ω, respectively. In the subsequent use of the finite element method, the weighted residuals of Equations (26) and (27) can be written as

(32) $$R_{i}^{1}=\int_{\Omega }w_{1}\left(\alpha^{2}\frac{\partial^{2}\psi }{\partial x^{2}}+\frac{\partial^{2}\psi }{\partial y^{2}}+\omega \;\right)d\Omega ,$$

(33) $$R_{i}^{2}=\int_{\Omega }w_{2}\left[Re\left(\frac{\partial \psi }{\partial y}\frac{\partial \omega }{\partial x}-\frac{\partial \psi }{\partial x}\frac{\partial \omega }{\partial y}\right)-\left(\alpha^{2}\frac{\partial^{2}\omega }{\partial x^{2}}+\frac{\partial^{2}\omega }{\partial y^{2}}\right)+\frac{\omega }{K}-M^{2}\frac{\partial^{2}\psi }{\partial y^{2}}\right]d\Omega ,$$

where $w_{1}$ and $w_{2}$ are weight functions and dΩ=dψdω. Simplifying Equations (32) and (33), the weak formulation yields the following equations:

(34) $$R_{i}^{1}=\int_{\Omega }\left(\alpha^{2}\frac{\partial \psi }{\partial x}\frac{\partial w_{1}}{\partial x}+\frac{\partial \psi }{\partial y}\frac{\partial w_{1}}{\partial y}\right)d\Omega -\int_{\Omega }w_{1}\omega \;d\Omega -\int_{\Omega }w_{1}\frac{\partial \psi }{\partial n}\;d\Gamma ,$$

(35) $$R_{i}^{2}=Re\int_{\Omega }w_{2}\left(\frac{\partial \psi }{\partial y}\frac{\partial \omega }{\partial x}-\frac{\partial \psi }{\partial x}\frac{\partial \omega }{\partial y}\right)d\Omega +\int_{\Omega }\left(\alpha^{2}\frac{\partial \omega }{\partial x}\frac{\partial w_{2}}{\partial x}+\frac{\partial \omega }{\partial y}\frac{\partial w_{2}}{\partial y}\right)d\Omega +\int_{\Omega }w_{2}\frac{\omega }{K}d\Omega \;+M^{2}\int_{\Omega }\frac{\partial \psi }{\partial y}\frac{\partial w_{2}}{\partial y}d+\int_{\Gamma }w_{2}\frac{\partial \omega }{\partial n}\;d\Gamma -M^{2}\int_{\Gamma }w_{2}\frac{\partial \psi }{\partial n}\;d\Gamma ,$$

where Ω denotes the the area integral for every element and Γ is the boundary for every element. Now, *Re* = 5 using Equation (31) in Equations (34) and (35), we get

(36) $$R_{i}^{1}=\sum_{i=1}^{n}\int_{\Omega }\left(\alpha^{2}\frac{\partial N_{i}}{\partial x}\frac{\partial N_{K}}{\partial x}+\frac{\partial N_{i}}{\partial y}\frac{\partial N_{K}}{\partial y}\right)\psi_{i}d\Omega -\overset{n}{\underset{i=1}{\sum }}\int_{\Omega }N_{i}N_{k}\omega_{i}d\Omega -\int_{\Gamma }N_{k}d\Gamma$$

(37) $$R_{i}^{2}=Re\;\sum_{i=1}^{n}\int_{\Omega }N_{k}\left(\frac{\partial N_{i}}{\partial y}\frac{\partial N_{i}}{\partial x}+\frac{\partial N_{i}}{\partial x}\frac{\partial N_{i}}{\partial y}\right)\psi_{i}\omega_{i}d\Omega \;+\;\sum_{i=1}^{n}\int_{\Omega }\left(\alpha^{2}\frac{\partial N_{i}}{\partial x}\frac{\partial N_{K}}{\partial x}+\frac{\partial N_{i}}{\partial y}\frac{\partial N_{K}}{\partial y}\right)\omega_{i}d\Omega +\frac{1}{K}\sum_{i=1}^{n}\int_{\Omega }N_{k}N_{i}\omega_{i}d\Omega \;+M^{2}\sum_{i=1}^{n}\int_{\Omega }\frac{\partial N_{i}}{\partial y}\frac{\partial N_{K}}{\partial y}d\Omega +\;\int_{\Gamma }N_{k}d\Gamma -M^{2}\int_{\Gamma }N_{k}d\Gamma$$

The assembled global matrix takes the form

(38) KA=F.

Equation (38) leads to a system of algebraic equations which occur non-linearly and can be solved by any iterative method. In the present investigation the well-known Newton Raphson method is used to solve the given system. The elaborated procedure is iterative to the desired convergence rate.

Peristaltic waves produced on the walls induce periodic flow. Due to this, we can evaluate pressure only on the dominant middle part of the channel, which is equal to one wavelength. To evaluate pressure, the expression in dimensionless form is given by

(39) $$\frac{\partial p}{\partial x}=Re\left\{\frac{\partial^{2}\psi }{\partial y^{2}}\frac{\partial \psi }{\partial x}-\frac{\partial^{2}\psi }{\partial x\partial y}\frac{\partial \psi }{\partial y}\right\}-\frac{\partial \omega }{\partial y}-\left(M^{2}+\frac{1}{K}\right)\left(\frac{\partial \psi }{\partial y}+1\right),$$

(40) $$\frac{\partial p}{\partial y}=\alpha^{2}Re\left\{\frac{\partial^{2}\psi }{\partial x^{2}}\frac{\partial \psi }{\partial y}-\frac{\partial^{2}\psi }{\partial x\partial y}\frac{\partial \psi }{\partial x}\right\}+\alpha^{2}\frac{\partial \omega }{\partial x}+\frac{\alpha^{2}}{K}\frac{\partial \psi }{\partial x}.$$

The pressure rise in the moving frame of reference is well defined as

(41) $$\Delta P_{\lambda }=\int_{0}^{\lambda }\frac{dp}{dx}dx.$$

## 4. Validation of Numerical Results

**Figure 2:**
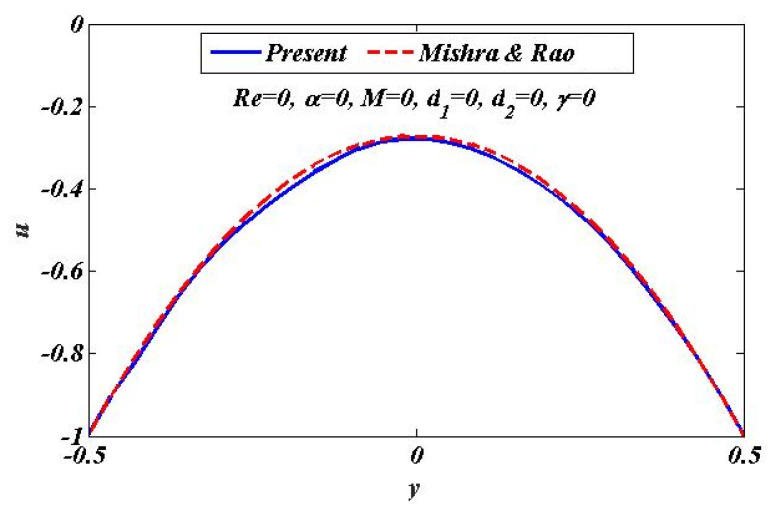
Comparative analysis for results with Mishra and Rao [33] for velocity distribution in limitation of *Re* = 0, *α* = 0, *M* = 0, *d*_1_ = 0, *d*_2_ = 0, and *γ* = 0.

This section is to check the validity of our present investigation by making a comparison between our obtained numerical results and the result of Mishra and Rao [33] in the limiting case, i.e., using the assumptions of lubrication theory in our present investigation, that is, $Re=0,\;\alpha =0,\;M=0,\;d_{1}=0,\;d_{2}=0$, and γ=0. The obtained solutions in the investigation by Mishra and Rao [33] are analytic. The velocity distribution based on our present numerical solutions is compared with that of Mishra and Rao [33] by using plots. It can be clearly observed from Figure 2 that in the limit of  γ=0,M=0 and $d_{1}=0,\;d_{2}=0,$ our calculated results have a remarkably good convergence rate with the results of Mishra and Rao [33]. Thus, it is indicated that the current study will be very useful for further research.

Analysis of the numerical results is presented in this section. Plots of velocity, streamlines, and pressure gradient are obtained and elaborated. Detailed discussion of the trapping of bolus is also presented. The effect of other pertinent parameters such as Reynolds number Re, Hartman number M, wave number α, and phase difference ϕ is also described.

### 4.1. Velocity Profile

**Figure 3:**
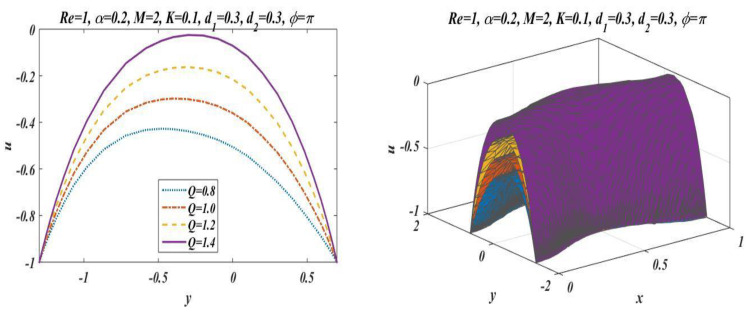
Velocity change for *Q* with *Re* = 1, *α* = 0.2, *M* = 2, *K* = 0.1, *d*_2_ = 0.3, *d*_1_ = 0.3 and *ϕ* = π.

**Figure 4:**
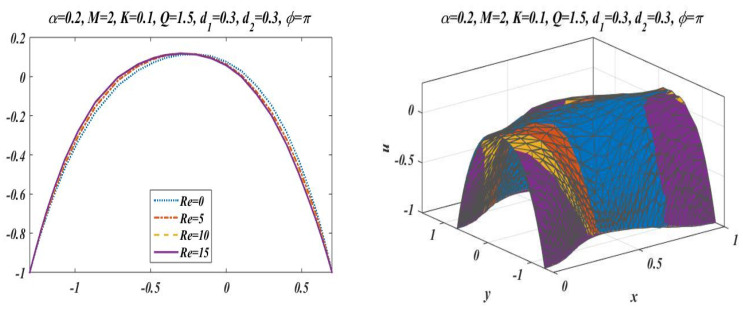
Variation in velocity for *Re* with *Q* = 1.5, *α* = 0.2, *M* = 2, *K* = 0.1, *d*_1_ = 0.3, *d*_2_ = 0.3 and *ϕ* = π.

**Figure 5:**
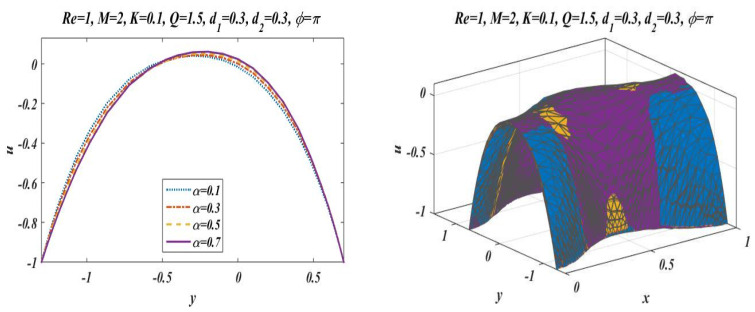
Velocity profile for *α* with *Re* = 1, *Q* = 1.5, *M* = 2, *K* = 0.1, *d*_1_ = 0.3, *d*_2_ = 0.3 and *ϕ* = π.

**Figure 6:**
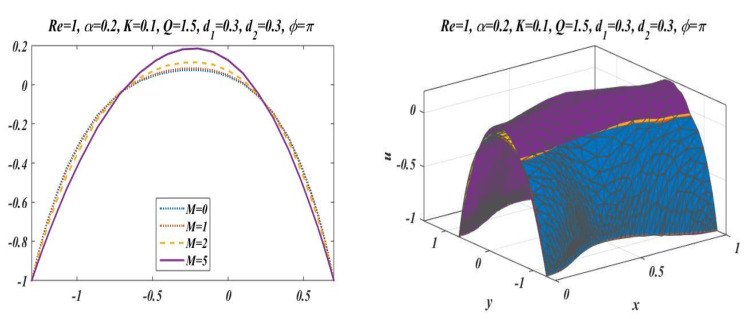
Velocity profile for *M* with *Re* = 1, *α* = 0.2, *Q* = 1.5, *K* = 0.1, *d*_1_ = 0.3, *d*_2_ = 0.3 and *ϕ* = π.

**Figure 7:**
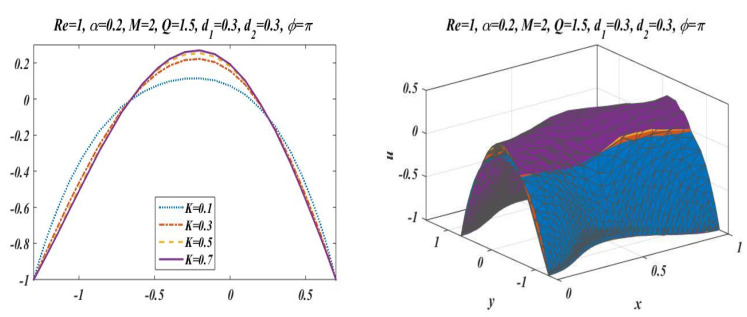
Velocity profile for *K* with *Re* = 1, *α* = 0.2, *M* = 2, *Q* = 1.5, *d*_1_ = 0.3, *d*_2_ = 0.3 and *ϕ* = π.

This subsection is presented here to discuss the behavior of velocity of fluid flowing through an asymmetric channel peristaltically. For this reason, a graph of longitudinal-velocity at cross section x=0 is plotted for various changing values of mean flow rate Q, Reynolds number Re, Hartmann Number M, porosity of porous media K, and wave number α in Figure 3, Figure 4, Figure 5, Figure 6 and Figure 7. From these plots it can be clearly concluded that the velocity of fluid on the walls of the channel vanishes due to no-slip conditions on the boundary. Figure 3 is designed to scrutinize the vibrational effects of mean flow rate Q for fixed values of other involved parameters. Time-mean flow rate measures the quantity of fluid passing through cross-sectional area of the channel. As we increase the value of time-mean flow rate Q, it means that more fluid is moving through the cross section, which means that the velocity is going to be increased. Figure 3 illustrates that increasing values of mean flow rate with rapid augmentation in the velocity profile are observed in the middle part and at the sides. The Reynolds number can be clearly defined as the ratio between inertial and viscous forces, so enhancing the value of Re leads to a decrease in viscous forces, which result in a decrease in opposition offered to the fluid. It can be seen from Figure 4 that by enhancing the value of the Reynolds number the longitudinal velocity is enhanced near the upper wall of the peristaltic channel, but reverse behavior is seen near the lower wall and mixed performance is seen in the middle part of the channel. Figure 5 is plotted to elaborate the effects of wave number for diverse values of α with static values of other parameters, i.e., $Re=1,\;Q=1.5,\;M=2,\;d_{1}=0.3,\;d_{2}=0.3,\;K=0.1\;,$ and ϕ=π. It shows that an increase in α results in a decline in the velocity near to the upper wall, and an increase in velocity of fluid is observed near the lower wall. Figure 6 and Figure 7 elaborate the changing effects of the Hartmann number M and porosity parameter K by keeping the other parameters fixed. It is evident that with an enhancement in the porosity parameter and magnetic field, an increase in velocity is observed in the central part of the peristaltic channel but velocity decreases near the walls. It may be concluded from Figure 6 and Figure 7 that the Hartmann number and porosity parameter are the best way to control the velocity of peristaltic flow.

### 4.2. Pressure Distribution

**Figure 8:**
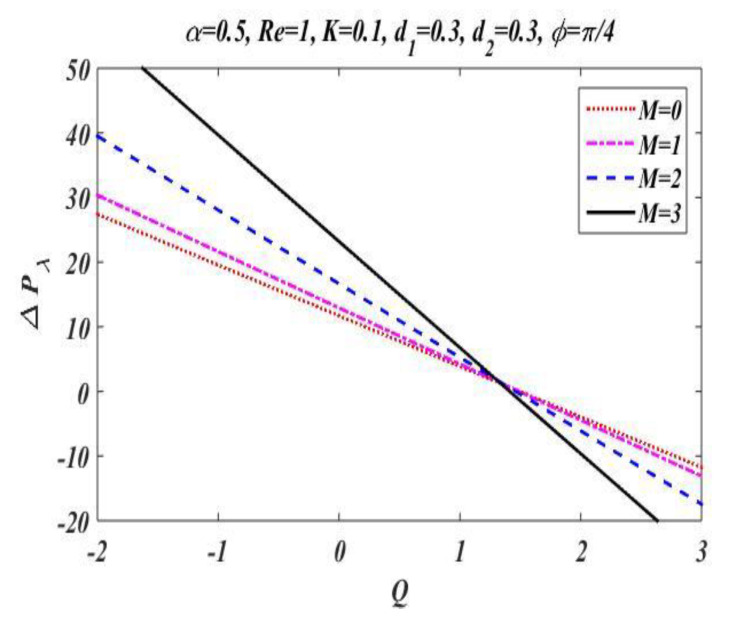
Pressure gradient for *M*.

**Figure 9:**
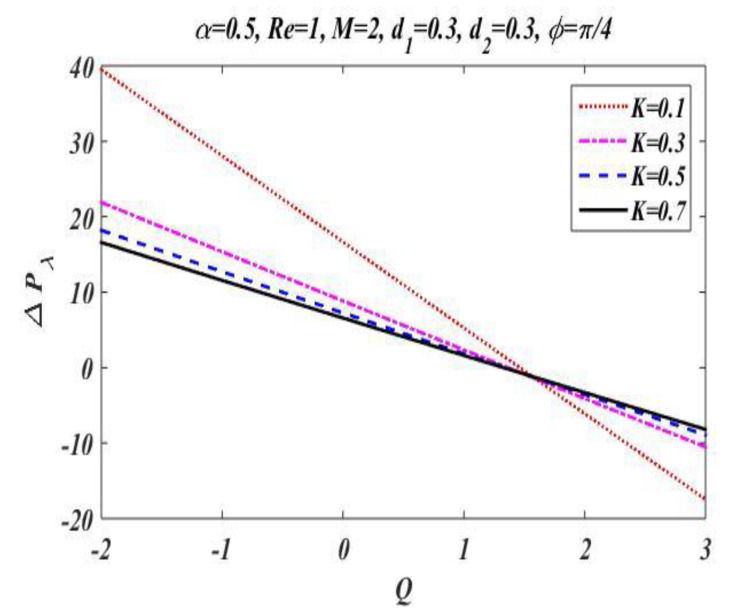
Pressure gradient for *K*.

**Figure 10:**
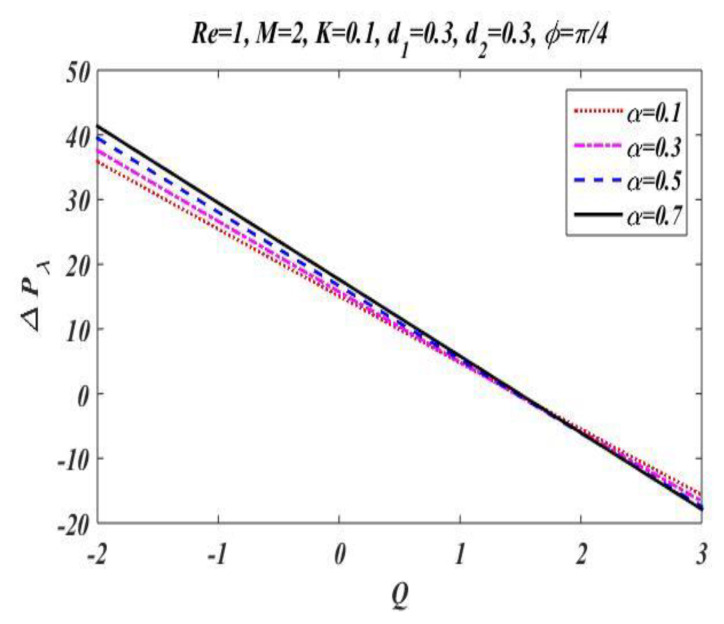
Pressure gradient for *α*.

**Figure 11:**
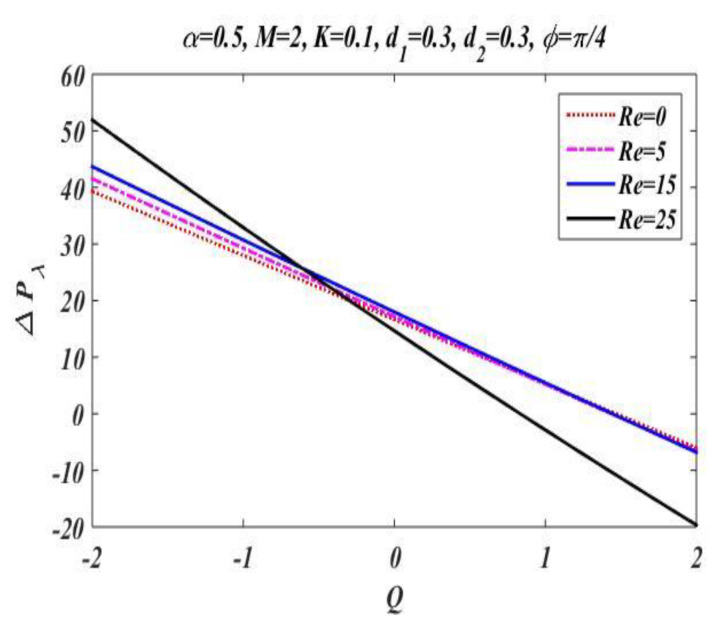
Pressure gradient for *Re*.

**Figure 12:**
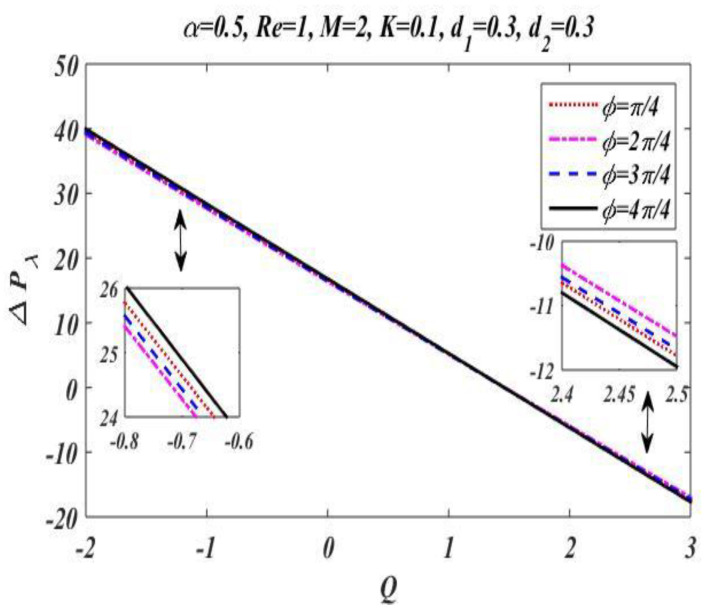
Pressure gradient for *ϕ*.

In the peristaltic phenomena, the fluid flows due to the waves which are produced on the walls of the channel. These waves produce a pressure which causes fluid to flow forward. This subsection is devoted to discussing the effect of involved pertinent parameters on pressure-rise per wavelength by changing the value of one parameter while keeping the others fixed. The variation effects of the Hartmann number M, Reynolds number Re, wave number α, porosity parameter K, and phase difference ϕ on pressure gradient is particularized in Figure 8, Figure 9, Figure 10, Figure 11 and Figure 12 by plotting the graph in the positive pumping region only. It is perceived that pressure increases with a rise in Hartmann number M, wave number, Reynolds number, and phase difference; additionally, a lessening in pressure is predicted in the augmented region. In contrast to the Hartmann and wave numbers, it is seen that pressure rise per wavelength declines with increase in the value of porosity parameter K in the peristaltic pumping region.

### 4.3. Trapping of Bolus

**Figure 13:**
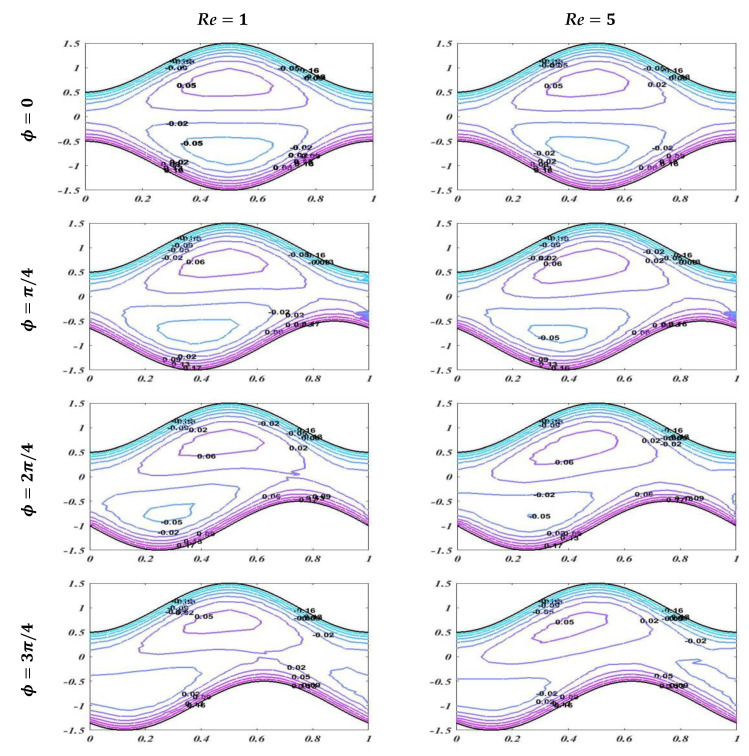

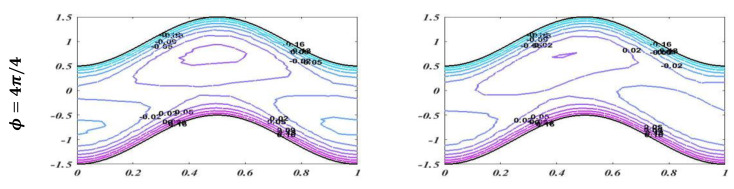
Streamlines with diverse phase shift for *Re* = 1 and *Re* = 5 with static values of other involved variables *α* = 0.2, *K* = 0.1, *M* = 1, *d*_1_ = *d*_2_ = 0.5, and *Q* = 1.6.

**Figure 14:**
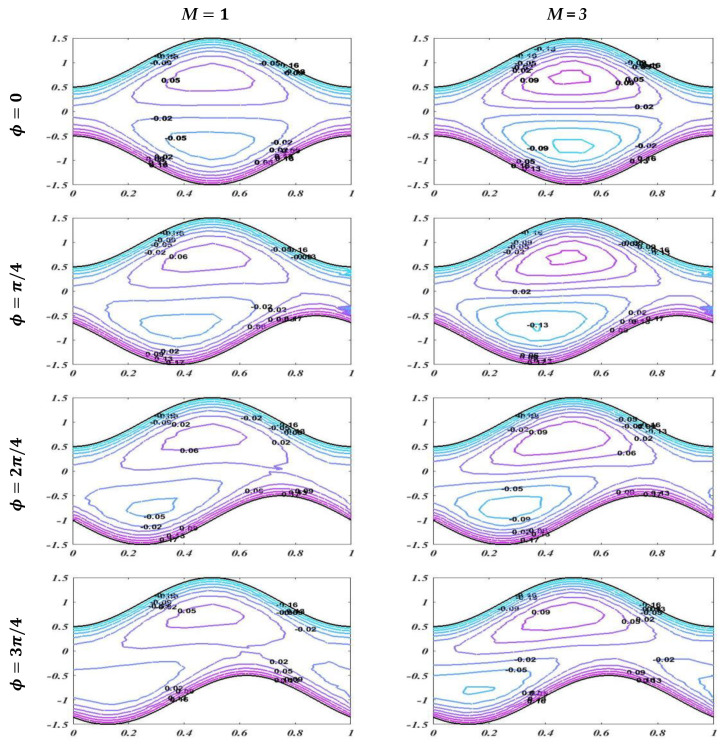

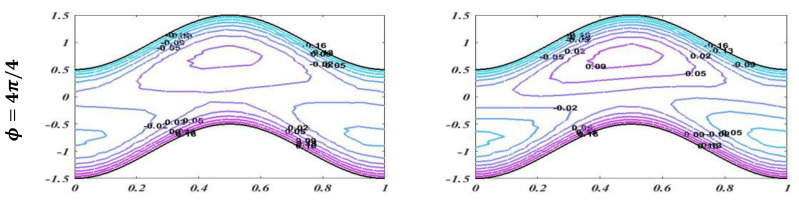
Streamlines with diverse Hartmann numbers for *M* = 1 and *M* = 3 with static values of other involved variables *α* = 0.2, *Re* = 1, *K* = 0.1, *d*_1_ = *d*_2_ = 0.5, and *Q* = 1.6.

**Figure 15:**
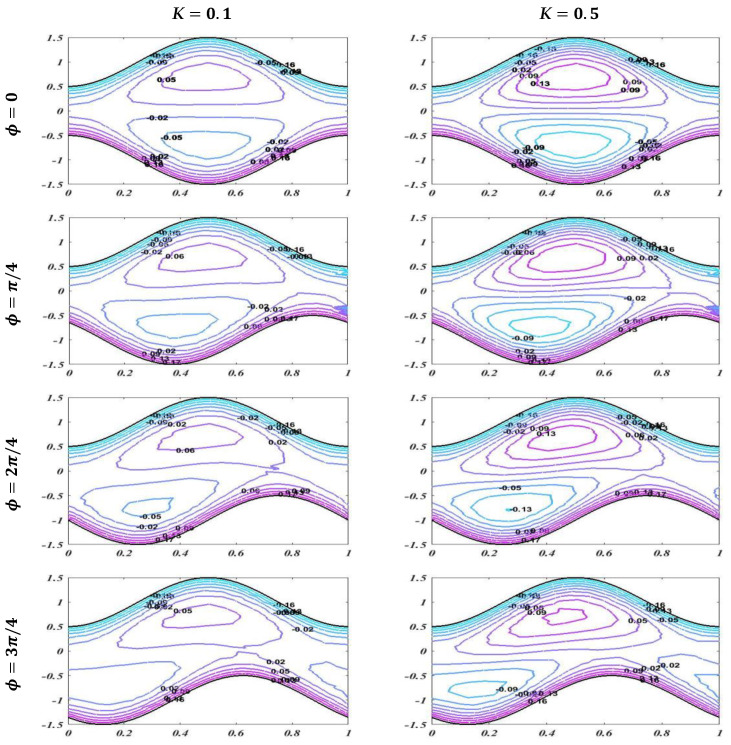

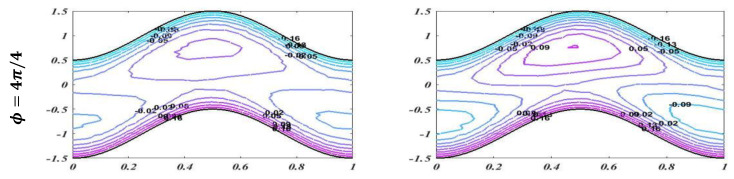
Streamlines with *K* = 0.1 and *K* = 0.3 when *α* = 0.2, *Re* = 1, *M* = 1, *d*_1_ = *d*_2_ = 0.5, and *Q* = 1.6.

The graph of streamlines is plotted in this subsection to describe the useful trapping phenomenon for different of values of Reynolds number Re, Hartmann number M, and porosity of porous media K for fixed values of other parameters with changing values of phase difference. Streamlines describes how the fluid is moving physically. Smooth streamlines correspond to smooth flow. As waves are generated on the wavy walls, these streamlines become more curved and this leads to enclosing a bolus which moves with the flow. In Figure 13, streamlines are plotted with an unlike phase shift for Re=1 and Re=5 with static values of other related parameters, i.e., $\alpha =0.2,K=0.1,\;M=1,\;d_{1}=d_{2}=0.5$, and Q=1.6. It is noted that when ϕ=0, it leads to symmetric channel, and there is no effect on bolus size by enhancing the value of the Reynolds number. However, when ϕ=π/4,2π/4,3π/4, this leads to an asymmetric channel. It should be noted that there is a slight change in bolus size, but as the asymmetry of the channel increases, it cause less retardation in the flow. Figure 14 and Figure 15 show that increasing the values of the applied magnetic field and porosity parameter leads to an increase in the size and number of boluses for both symmetric and asymmetric channels, which causes more resistance and decreases the fluid flow. It is also observed that in case of enhancement of K, the number of boluses is more than in the case of an increase in the Hartmann number M.

## 5. Conclusions

In the present work the investigation of peristaltic transport is carried out in an asymmetric channel under an applied magnetic field. The numerical simulations are carried out using the finite element method. Moreover, lubrication theory is not used in this study. The present work leads to the following conclusions:

The velocity is enhanced by increasing time-mean flow rate Q and Reynolds number Re but declines with an increase in the value of wave number α. 

It is seen that by increasing the Hartmann number and porosity parameter K, the velocity of peristaltic flow is enhanced, but pressure distribution declines with increasing porosity parameter.

Increasing the value of the Reynolds number leads to enhancement in the velocity and pressure of the flow. However, there is a slight change in the size of the bolus.

The streamlines illustrate that the number of boluses is increased by enhancing the Hartmann number and porosity parameter.

It is concluded that magnetic field and porosity are the best factors with which to control the velocity, pressure, and boluses in the peristaltic flow.

## Data Availability

All the data are clearly mentioned in the manuscript.
